# Subtyping of Type 1 Diabetes as Classified by Anti-GAD Antibody, IgE Levels, and *Tyrosine kinase 2* (*TYK2*) Promoter Variant in the Japanese

**DOI:** 10.1016/j.ebiom.2017.08.012

**Published:** 2017-08-12

**Authors:** Keiichiro Mine, Kanako Hirakawa, Shiori Kondo, Masae Minami, Akira Okada, Nobutaka Tsutsu, Yasushi Yokogawa, Yumi Hibio, Fumiko Kojima, Shuji Fujimoto, Hironori Kurisaki, Keizo Anzai, Yasunobu Yoshikai, Seiho Nagafuchi, the West Japan Pathogenesis of Diabetes Study Group

**Affiliations:** a Division of Host Defense, Medical Institute of Bioregulation, Kyushu University, 3-1-1, Maidashi, Higashi-ku, Fukuoka, Japan; b Department of Medical Science and Technology, Graduate School of Medical Sciences, Kyushu University, 3-1-1, Maidashi, Higashi-ku, Fukuoka, Japan; c Matsuyama Red Cross Hospital, 1, Bunkyo-machi, Matsuyama-shi, Ehime, Japan; d Minami Masae Naika Clinic, 1-4-6, Heiwa, Minami-ku, Fukuoka, Japan; e Okada Naika Clinic, 7-8-8, Hakozaki, Higashi-ku, Fukuoka, Japan; f Department of Diabetes and Metabolism, Fukuoka Red Cross Hospital, 3-1-1, Minami-ku, Fukuoka, Japan; g Yokogawa Naika Clinic, 5-7-7, Tenjin, Chuo-ku, Fukuoka, Japan; h Center for Clinical Laboratory Examination, Fukuoka Medical Association, 1-6-9, Sawara-ku, Fukuoka, Japan; i Department of Hepatology, Diabetes and Endocrinology, Faculty of Medicine, Saga University, 5-1-1, Nabeshima, Saga, Japan

**Keywords:** Type 1 diabetes (T1D), Anti-GAD, IgE, *Tyrosine kinase 2* (*TYK2*), Th1/Th2, Virus, T1D, Type 1 diabetes, Th1, type 1 T helper, *TYK2*, *Tyrosine kinase 2*, HIES, hyper-IgE syndrome, Th2, type 2 T helper, Anti-GAD Ab, anti-glutamic acid decarboxylase antibody, YH, young healthy controls, OR, odds ratio, CI, confidence interval

## Abstract

**Objective:**

Type 1 diabetes (T1D) is known to be caused by Th1 cell-dependent autoimmunity. Recently, we reported that *TYK2* promoter variant serves as a putative virus-induced diabetes susceptibility gene associated with deteriorated interferon-dependent antiviral response. *TYK2* is also related to HIES, that is, Th2 cell-dependent. Therefore, *TYK2* promoter variant may be also associated with the pathogenesis of T1D, modulating Th1/Th2 balance.

**Research Design and Methods:**

We assessed the association between anti- GAD Ab, IgE levels, and *TYK2* promoter variant among 313 T1D patients, 184 T2D patients, and 264 YH controls in the Japanese.

**Results:**

T1D patients had elevated IgE (median, 56.7 U/ml; *p* < 0.0001) compared with T2D patients (22.5 U/ml) and controls (43.3 U/ml). Contrary to our expectations, there was no correlation between *TYK2* promoter variant and IgE levels. We found that T1D could be subtyped as four groups based on anti-GAD Ab and IgE profile: Subtype 1, anti-GAD Ab positive and non-elevated IgE (47.0%); Subtype 2, anti-GAD Ab negative and non-elevated IgE (35.1%); Subtype 3, anti-GAD Ab positive and elevated IgE (10.9%); and Subtype 4, anti-GAD Ab negative and elevated IgE (7.0%). In Subtype 2, a significantly higher incidence was observed in T1D cases carrying the *TYK2* promoter variant (OR, 2.60; 95%CI, 1.03–6.97; *p* = 0.032), and also showing a flu-like syndrome at diabetes onset (OR, 2.34; 95%CI, 1.27–4.35; *p* = 0.003).

**Interpretation:**

Anti-GAD Ab and IgE profiling helps classifying T1D into four groups that recognize variable pathogenic bases of T1D.

## Highlights

• T1D can be subtyped into 4 groups based on anti-GAD Ab, IgE levels and *TYK2* promoter variant.
• Only one subtype, without anti-GAD Ab or elevated IgE, was associated with *TYK2* promoter variant and flu-like syndrome.
• Profiling of T1D by anti-GAD Ab and IgE levels is useful to realize the variable immuno-pathogenesis of T1D.

Since *TYK2* gene is associated with T1D and HIES, we assessed the association between anti-GAD Ab, IgE levels, and *TYK2* promoter variant in patients with T1D. Consequently, T1D can be subtyped into 4 distinct groups based on these clinical laboratory and genetic markers. Only those patients without anti-GAD Ab or elevated IgE were associated with *TYK2* promoter variant and with flu-like syndrome at the diabetes onset, suggestive of association with virus-induced diabetes. This study describes the usefulness of subtyping of T1D with variable immunological bases and provides a clue to delineate the pathogenesis of T1D.

## 1 Introduction

T1D is caused by extensive destruction of insulin-producing pancreatic beta-cells leading to absolute insulin deficiency, and the incidence has been increasing worldwide at a rate of 3% every year (American Diabetes Association, 2014, Atkinson et al., 2014, IDF Diabetes Atlas Seventh Edition 2015, 2015, Scully, 2012). The American Diabetes Association has proposed two classifications of T1D, immune-mediated (Type 1A) and idiopathic (Type 1B) (American Diabetes Association, 2014). The immune-mediated form of T1D results from cellular mediated autoimmune destruction of pancreatic beta-cells, has strong associations with HLA, and is characterized by the production of several autoantibodies including anti-insulin antibody (IAA), anti- GAD Ab, islet antigen 2 antibody (IA-2 Ab), anti-zinc transporter antibody (ZnT8 Ab), and historic anti-islet cell antibody (ICA Ab) (American Diabetes Association, 2014). It has been well established that T1D is mainly a Th1 cell-dependent autoimmune associated disease (Haskins and Cooke, 2011), while this assignment of Th cells has been largely based on precarious conditions in experimental animals that did not correctly reflect the delicate balance or the relative contribution of each Th subset throughout the disease (Azar et al., 1999). It was reported that Th2 cells may play a progressive role by accelerating autoimmunity due to production of Th2 cytokines (Azar et al., 1999). In contrast, the idiopathic form of T1D is strongly inherited, but there is no evidence of autoimmunity or HLA association (American Diabetes Association, 2014). Fulminant T1D in which a non-autoimmune process may associate with the onset was also reported as an important subtype in East Asia (Imagawa and Hanafusa, 2011). These observations imply that T1D patients possibly possess a delicate Th1/Th2 balance. Overall, it was suggested that T1D seems to include heterogeneous diseases whose pathogenic processes, immunologic basis, genetics, and phenotypic characteristics present marked variations (Atkinson et al., 2014, Kawasaki and Eguchi, 2004).

The importance of environmental factors for T1D onset has also been well documented (Atkinson et al., 2014, Coppieters et al., 2012, de Beeck and Eizirik, 2016). Viruses, as one of the environmental factors, particularly coxsackieviruses that belong to the genus enterovirus in the *Picornaviridae* family, have long been suspected to contribute to the T1D onset (Coppieters et al., 2012, de Beeck and Eizirik, 2016, Jun and Yoon, 2003). Multiple factors could interplay among enterovirus, immune system and host genes (Hober and Sauter, 2010), as enterovirus infection may lead to the activation of innate and adaptive immunity against pancreatic beta cells (Hober and Sauter, 2010). The mechanisms of beta-cell destruction by viruses have been reported: induced direct virolysis of beta-cells, local inflammatory responses, or virus infection triggering beta-cell specific autoimmunity, together leading to destruction of beta-cells (de Beeck and Eizirik, 2016, Jun and Yoon, 2003). The former situation seems to be the case of high dose encephalomyocarditis (EMC)-D virus (a picornavirus)-induced diabetes in inbred mice, which is an excellent animal model resembling fulminant T1D in humans (Shimada and Maruyama, 2004, Nagafuchi et al., 2013). Since intact innate anti-viral responses play a pivotal role in the protection against picornavirus infection (Takeuchi and Akira, 2009), it is suggested that innate immunity-associated genes are candidates for determining susceptibility to virus-induced diabetes (Kounoue et al., 2008, Nagafuchi et al., 2013). Consistently, some innate immunity associated genes have been reported as candidate genes for T1D. These include helicase C domain 1 (*IFIH1*) (or melanocyte differentiation antigen (*MDA*) 5), protein tyrosine phosphatase non-receptor type 2 (*PTPN2*), BTB domain and CNC homolog 2 (*BACH2*), and *TYK2* (de Beeck and Eizirik, 2016, Marroqui et al., 2015, Onengut-Gumuscu et al., 2015).

*TYK2* is a member of the Janus kinase (JAK) family, and plays an important role in signals of type 1 IFN and IL-12 to resist against microbial infections (Leitner et al., 2015, Shimoda et al., 2000). Genetically-determined alternations of IFN responses, including *TYK2* gene, are detrimental in immune and inflammatory disease such as T1D (Leitner et al., 2015, Jean-Baptiste et al., 2017). Recently, based on the discovery of natural mutations of *TYK2* gene as murine encephalomyocarditis (EMC)-D virus-induced diabetes susceptibility gene causing deteriorated type 1 interferon (IFN) response (Izumi et al., 2015), we could show that “*TYK2* promoter variant” in Japanese subjects is associated with an increased risk of T1D (Nagafuchi et al., 2015). The prevalence rate of *TYK2* promoter variant is high in overall T1D (9.6%; OR, 2.4: *p* = 0.012), most highly in T1D associated with flu-like syndrome at diabetes onset (13.7%; OR, 3.6: *p* = 0.005), and anti-GAD Ab negative T1D (12.8%; OR, 3.3: *p* = 0.0021), compared with age- and sex-matched healthy controls (4.2%) (Nagafuchi et al., 2015). These results suggested that *TYK2* promoter variant may serve as a virus-induced T1D susceptibility gene, possibly due to reduced type 1 IFN response (Nagafuchi et al., 2015), but not Th1 cell-dependent autoimmunity. Consistently, it was reported that *Tyk2*-mediated signaling was not essential for the development of Th1 cell (Hashiguchi et al., 2014). *TYK2* promoter variant serves as a risk not only in T1D but also in T2D, suggesting that *TYK2* promoter variant is associated with an overall risk for diabetes (Nagafuchi et al., 2015). It has also been reported that *TYK2* gene is closely linked with HIES, that is, Th2 cell-dependent immune response-associated disease (Minegishi et al., 2006). *TYK2* deficiency is a type of primary immunodeficiency displaying the phenotype of the autosomal recessive HIES, and is likely to account for the phenotype of impaired Th1 differentiation and accelerated Th2 differentiation (Minegishi et al., 2006). *TYK2* gene is thus closely related to immunologic condition and therefore, the *TYK2* promoter variant may possibly be associated with the pathogenesis of T1D modulating Th2 cell-dependent immunologic responses.

In the present study, we focused on *TYK2* gene as a susceptibility gene for both T1D and HIES, and assessed the association among anti-GAD Ab, IgE levels, and *TYK2* promoter variant in diabetic patients. Here we report the immunological bases on which T1D forms may be classified into four subtypes among T1D by simple clinical markers.

## 2 Materials and Methods

### 2.1 Subjects

We studied 313 patients with T1D, 184 patients with T2D and 264 young non-diabetic subjects (YH) (these are not age-matched with the T1D and T2D cohorts - see also Table 1) in the western Japan region. These subjects partly provided the same samples which were studied in our previous article (Nagafuchi et al., 2015), and sample size of T1D has been shown to be appropriate for the T1D case control study. For comparison, estaimated suitable number of patients with T2D and Young Healthy Controls were chosen to be applicable for appropriate statistical analysis in case-control study by computer. The clinical profiles of patients studied are presented in Table 1. Among the 313 patients with T1D, 76 patients were associated with flu-like syndrome at the onset. Symptoms of flu-like syndrome include fever, chills, sore throat, muscle and joint aches, poor appetite, diarrhea, cough, and fatigue, suggestive of certain viral infections not limited to enterovirus infection. Those patients with clinical features such as tonsillitis, pneumonia, or urinary tract infection associated with neutrophilia, suggestive of bacterial origin, were not regarded as patients with flu-like syndrome and were excluded from the group. The study was conducted in accord with case-control studies of STROBE statement. Since it was reported that there was a peak of IgE levels in the group of 19 to 21 years old (De Amici and Ciprandi, 2013), we selected YH as a control group. Patients were designated as T1D if fasting C-peptide was < 0.5 ng/ml with insulin-dependent condition (IDDM), or as T2D if fasting blood glucose levels were higher than 126 mg/dl and HbA1c levels exceeded 6.5% with non-insulin-dependent status (NIDDM). In Japanese T1D patients, positivity of anti-GAD Ab is reported to be 60–70% (Kawasaki and Eguchi, 2004). The individuals had no clinical sign of allergy. The study was conducted according to the guidelines for human study and was approved by the ethical committee of the Kyushu University Graduate School of Medical Sciences (No.433-00 and 433-01). Written informed consent was obtained from all subjects including T1D, T2D, and YH involved in this study.

**Table 1:** Characteristics of patients with T1D, T2D, and young healthy controls.

Characteristics	Type 1 diabetes	Type 2 diabetes	Young healthy^g^
Number	313	184	264
Age - yr (range)	40.7 ± 17.3 (7–83)	65.8 ± 11.4 (20–88)	21.1 ± 2.3 (19–36)
TYK2 promoter variant (%)	29 (9.3)	11 (6.0)	19 (7.2)
Heterozygous polymorphism (%)	28 (8.9)	10 (5.4)	18 (6.8)
Homozygous polymorphism (%)	1 (0.3)	1 (0.5)	1 (0.4)
*p*-value (95%CI)	0.23^d^ (0.75–3.65)		–
Flu-like syndrome at diabetes onset^a^ (%)	76 (24.3)	NA^e^	NA
% Anti-GAD Ab positive^b^ (All, *n* = 313)	58	NT^f^	NT
Without flu-like syndrome (*n* = 237)	61.6	NA	NA
With flu-like syndrome (*n* = 76)	44.7	NA	NA
HbA1c (%)^c^	8.5 ± 2.2	7.5 ± 1.3	NT
BMI (kg/m^2^)	21.7 ± 3.1	23.7 ± 4.2	21.0 ± 2.9
Age at diabetes onset (range)	27.1 ± 17.9 (0–73)	NA	NA
Disease duration (range)	14.0 ± 10.7 (0–59)	NA	NA

Values are means ± standard deviation.

a Symptoms of flu-like syndrome include fever, chills, sore throat, muscle and joint aches, poor appetite, diarrhea, cough, and fatigue, suggestive of certain viral infections.

b Anti-GAD Ab: anti-glutamic acid decarboxylase antibody (≧ 1.5 U/ml).

c HbA1c (%) was expressed as National Glycohemoglobin Standardization Program (NGSP) value.

d The prevalence of *TYK2* promoter variant was not significant differences in T1D vs T2D.

e NA: not available.

f NT: not tested.

g Young healthy: not age-matched.

#### 2.1.1 *TYK2* Promoter Variant Genotyping

*TYK2* promoter variant was assessed by PCR analysis, as described previously (Nagafuchi et al., 2015). Genotyping had been performed to detect the putative promoter region, 1.3 kb upstream of start codon, of the TYK2 gene. TYK2 sequence reference was NCBI Reference Sequence: NG_007872.1. TYK2 gene located at 19p13.2. SNPs in *TYK2* promoter variant are as follows: NC_000019.10:g.10381501_10381502delAC (rs number is under submission), NC_000019.10:g.10380676T > G (rs2304259), NC_000019.10:g.10380572T > C (rs17000730), NC_000019.10:g.10380511C > T (rs17000728), NC_000019.10:g.10380510C > T (rs2304258). Because these polymorphisms at the upstream and 5’UTR of *TYK2* gene were in complete linkage disequilibrium (Lewontin's D' = 1), the haplotype was named *TYK2* promoter variant (Nagafuchi et al., 2015).

#### 2.1.2 Anti-GAD Ab, Anti-IA-2 Ab and IgE Analysis

Anti-GAD Ab were determined by radioimmunoassay (RIA). Anti-GAD Ab levels higher than 1.5 U/ml were considered as positive. Anti-IA-2 Ab were determined by radioimmunoassay (RIA). Anti-IA-2 Ab levels higher than 0.4 U/ml were considered as positive. We found that only ten were anti-IA-2 Ab positives; six were also anti-GAD Ab positive among 181 anti-GAD Ab positives, and four were anti-GAD Ab negative among 132 anti-GAD negative patients with T1D. The numbers of anti-IA-2 Ab positives were so few and therefore, to avoid complexity, the anti-IA-2 Ab data were not involved in the further analysis of this study. IgE levels in T1D patients, T2D patients, and YH was measured with a human IgE enzyme-linked immunosorbent assay (ELISA) quantitation set (Bethyl Laboratories Inc. USA), according to the manufacturer's instructions. The levels of IgE data used in this study were the means of triplicate measurements. IgE levels higher than 170 U/ml were diagnosed as positive.

#### 2.1.3 Subtyping and Numbering of T1D Patients

T1D patients were classified into four subtypes by IgE levels (< 170 U/ml, low; ≧ 170 U/ml, high) and anti-GAD Ab (< 1.5 U/ml, negative; ≧ 1.5 U/ml, positive) profile. Numbering of subtypes was done according to the number of the patients.

### 2.2 Statistical Analysis

**Fig. 1:**
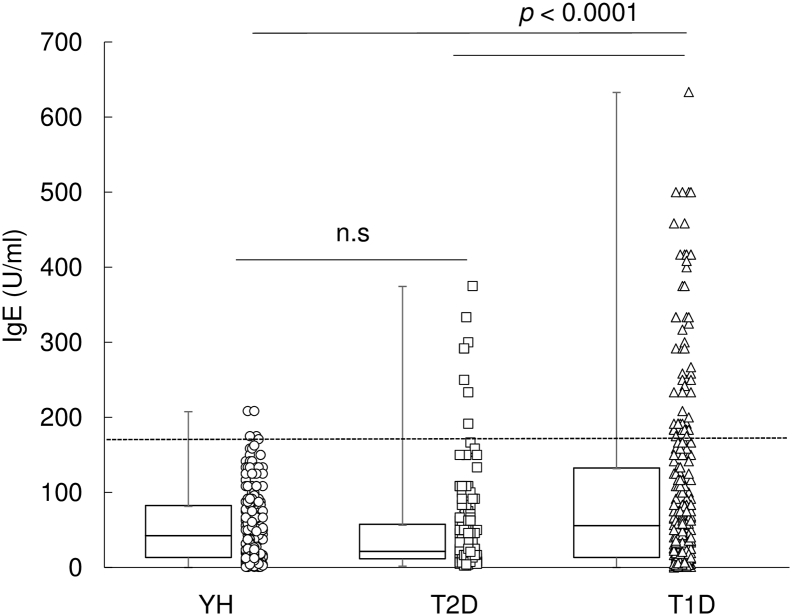
Elevated IgE in T1D patients. The levels of IgE in YH (*n* = 264), T2D patients (*n* = 184) and T1D patients (*n* = 313) were scattered and plotted as box plots, indicating lower quartile, median, and higher quartile, with whiskers representing the range of the remaining data points. Patients with T1D showed higher IgE levels (median, 56.7 U/ml; *p* < 0.0001) than T2D patients (median, 22.5 U/ml) or YH (median, 43.3 U/ml). The dotted line indicates the upper limit of the IgE normal range, 170 U/ml. n.s., not significant.

**Fig. 2:**
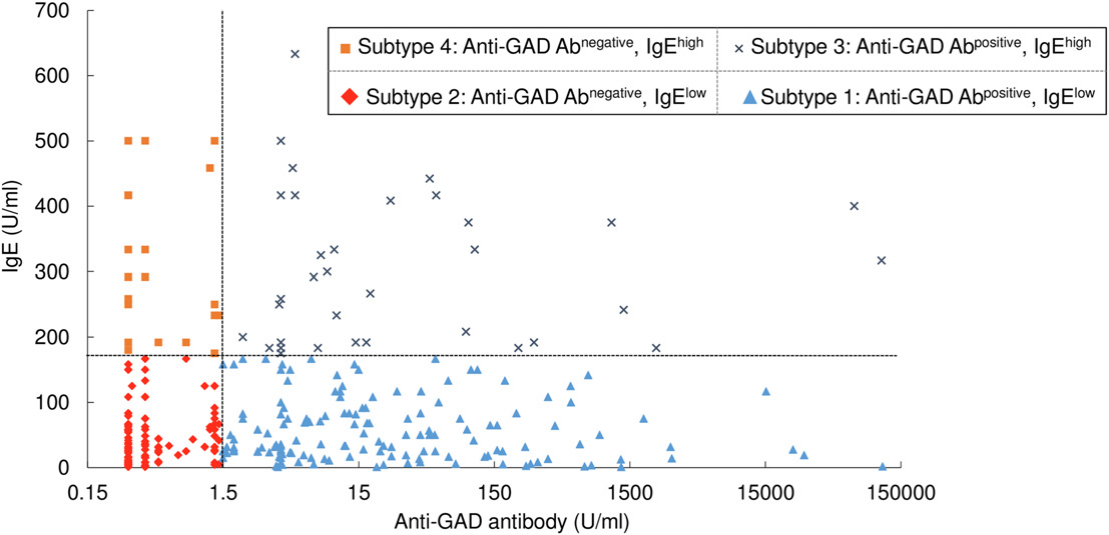
Four subtypes of T1D based on anti-GAD Ab and IgE profile. Total IgE levels plotted against anti-GAD Ab from patients with T1D (*n* = 313). Patients with T1D were classified into four subtypes by IgE levels (< 170 U/ml, low; ≧ 170 U/ml, high) and anti-GAD Ab (< 1.5 U/ml, negative; ≧ 1.5 U/ml, positive). Subtype 1 (blue triangle), anti-GAD Ab positive and IgE low (47.0%, *n* = 147); Subtype 2 (red rhomb), anti-GAD Ab negative and IgE low (35.1%, *n* = 110); Subtype 3 (gray cross), anti-GAD Ab positive and IgE high (10.9%, *n* = 34); and Subtype 4 (orange square), anti-GAD Ab negative and IgE high (7.0%, *n* = 22). There was no correlation between anti-GAD Ab and IgE levels (*r* = 0.060, *p* = 0.289). The vertical dotted line indicates the 1.5 U/ml value of anti-GAD Ab, and the horizontal dotted line indicates the 170 U/ml levels of IgE. The x axis is the logarithmic scale.

**Fig S1:**
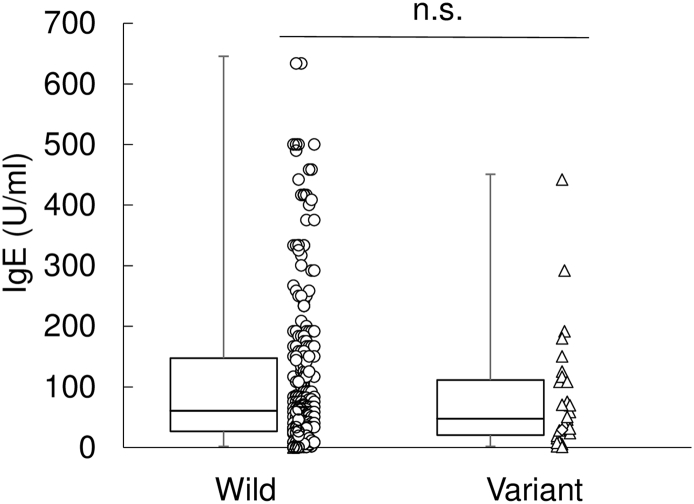
*TYK2* promoter variant does not affect IgE levels. Total IgE levels from T1D patients with wild type *TYK2* gene (*n* = 284) or *TYK2* promoter variant (*n* = 29) (wild, median, 58.3; variant, median, 45.8; *p* = 0.440). Data are scattered and plotted as box plots, indicating lower quartile, median, and higher quartile, with whiskers representing the range of the remaining data points. n.s., not significant.

**Fig S2:**
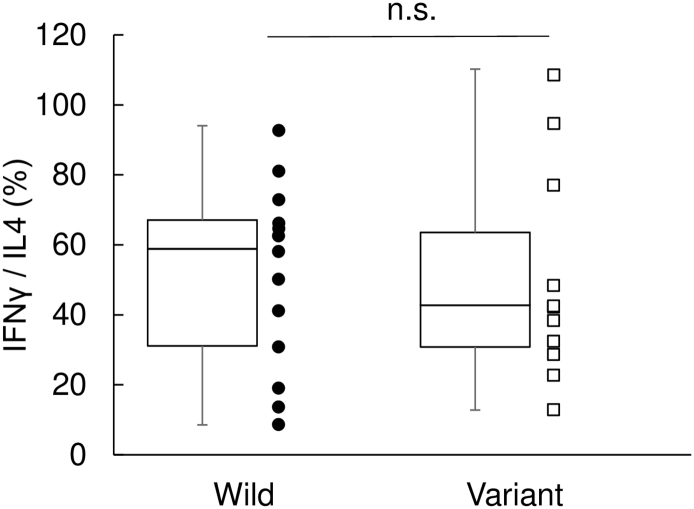
The distribution of T1D patients with flu-like syndrome at T1D onset according to anti-GAD Ab and IgE profile. Total IgE levels plotted against anti-GAD Ab from patients with flu-like syndrome at T1D onset (*n* = 76). Patients with flu-like syndrome at T1D onset were classified into four subtypes by IgE levels (< 170 U/ml, low; ≧ 170 U/ml, high) and anti-GAD Ab (< 1.5 U/ml, negative; ≧ 1.5 U/ml, positive). Blue triangle, anti-GAD Ab positive and IgE low (35.5%, *n* = 27) (belonging to Subtype 1); red rhomb, anti-GAD Ab negative and IgE low (50.0%, *n* = 38) (belonging to Subtype 2); gray cross, anti-GAD Ab positive and IgE high (9.2%, *n* = 7) (belonging to Subtype 3); and orange square, anti-GAD Ab negative and IgE high (5.3%, *n* = 4) (belonging to Subtype 4). There was no correlation between anti-GAD Ab and IgE levels (*r* = 0.195, *p* = 0.091). The vertical dotted line indicates the 1.5 U/ml value of anti-GAD Ab, and the horizontal dotted line indicates the 170 U/ml levels of IgE. The x axis is the logarithmic scale.

**Fig S3:**
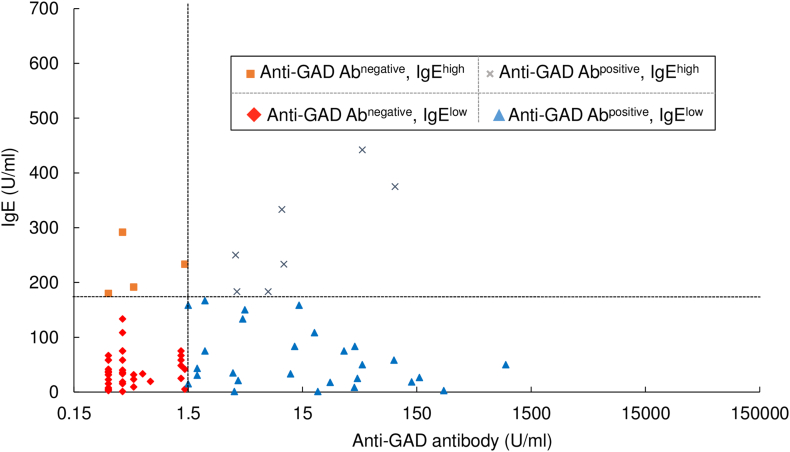
*TYK2* promoter variant did not associate with Th1/Th2 balance. Th1/Th2 balance from diabetic patients with wild type *TYK2* gene (*n* = 13) or *TYK2* promoter variant (*n* = 11) (wild, median, 58.0; variant, median, 42.2; *p* = 0.932). Data are scattered and plotted as box plots, indicating lower quartile, median, and higher quartile, with whiskers representing the range of the remaining data points. n.s., not significant.

Statistical analysis was performed in the statistical program R (http://cran.r-project.org). Data were analyzed by using: Fig. 1, and Fig. S1, Fisher's exact test, classified by the line of 170 U/ml (< 170 U/ml, low; ≧ 170 U/ml, high) for each group; Fig. 2 and Fig. S2, Spearman's rank correlation test; Fig. S3, Welch's *t*-test; Table 2 and Table S1, Fisher's exact test.

**Table 2:** Four subtypes of T1D based on anti-GAD Ab and IgE profile.

Subtype	Anti-GAD antibody (U/ml)	IgE (U/ml)	Number (%) (*n* = 313)	*TYK2* promoter genotype				Flu-like syndrome at diabetes onset^a^		
				Wild (*n* = 284)	Variant (%) (*n* = 29)	OR b (95%CI^c^)	*p*-value	Number (%) (*n* = 76)	OR (95%CI)	*p*-value
1	≧ 1.5	< 170	147 (47.0)	138	9 (6.1)	reference	reference	27 (18.4)	reference	reference
2	< 1.5	< 170	110 (35.1)	94	16 (14.5)	2.60 (1.03–6.97)	0.032	38 (34.5)	2.34 (1.27–4.35)	0.003
3	≧ 1.5	≧ 170	34 (10.9)	31	3 (8.8)	1.48 (0.244–6.40)	0.700	7 (20.6)	1.15 (0.383–3.09)	0.808
4	< 1.5	≧ 170	22 (7.0)	21	1 (4.5)	0.731 (0.016–5.77)	1.00	4 (18.2)	0.988 (0.225–3.34)	1.00

a Symptoms of flu-like syndrome include fever, chills, sore throat, muscle and joint aches, poor appetite, diarrhea, cough, and fatigue, suggestive of certain viral infections.

b OR, odds ratio.

c CI, confidence interval.

## 3 Results

### 3.1 Comparison of IgE Levels in Patients With T1D, T2D and YH

*TYK2* gene plays a key mediator for both T1D and HIES, whereas these diseases were reported to have different Th cell-dependent background (Minegishi et al., 2006, Nagafuchi et al., 2015). Moreover, IgE levels have been reported as a potential risk factor of diabetes (Wang et al., 2011), and T1D may possibly be associated with a risk of self-reported presence of IgE-mediated allergies (Klamt et al., 2015).

Therefore, we investigated the IgE levels of 313 patients with T1D, 184 patients with T2D, and 264 YH to reveal the significance of IgE levels in diabetic patients. Consequently, T1D patients showed higher IgE levels (median, 56.7 U/ml; *p* < 0.0001) than T2D patients (median, 22.5 U/ml) or YH (median, 43.3 U/ml) (Fig. 1), suggesting that elevated IgE in T1D patients have distinct clinical significance.

### 3.2 Subtyping of T1D based on Anti-GAD Ab, IgE levels and *TYK2* promoter variant

Because anti-GAD Ab has been shown to be an excellent and major autoantibody of T1D, and is also known to reflect Th1 cell-dependent immune destruction of pancreatic beta-cells (American Diabetes Association, 2014), we chose anti-GAD Ab as a Th1 marker. We determined the association of IgE levels and anti-GAD Ab in T1D patients to reveal immune condition in T1D patients. As a result, we could subtype T1D patients into four groups based on anti-GAD Ab (< 1.5 U/ml, negative; ≧ 1.5 U/ml, positive) and IgE (< 170 U/ml, low; ≧ 170 U/ml, high) profile (Fig. 2). These subtypes were named as Subtype 1, anti-GAD Ab positive and non-elevated IgE (*n* = 147, 47.0%); Subtype 2, anti-GAD Ab negative and non-elevated IgE (*n* = 110, 35.1%); Subtype 3, anti-GAD Ab positive and elevated IgE (*n* = 34, 10.9%); and Subtype 4, anti-GAD Ab negative and elevated IgE (*n* = 22, 7.0%) (Table 2). Subtype 1 seems to be classical T1D with Th1 cell-dependent autoantibody positivity, and therefore, we used Subtype 1 as a reference. Among these subtypes, *TYK2* promoter variant had a significantly high incidence in Subtype 2: anti-GAD Ab negative and non-elevated IgE (OR, 2.60; 95%CI, 1.03–6.97; *p* = 0.032) (Table 2). Furthermore, prevalence of flu-like syndrome at diabetes onset was also significantly high in Subtype 2 (OR, 2.33; 95%CI, 1.27–4.35; *p* = 0.003) (Table 2). These observations taken together imply the following interpretations regarding these four subtypes: Subtype 1, classical Th1 cell-activated autoimmune T1D; Subtype 2, without anti-GAD Ab and non-elevated IgE in association with *TYK2* promoter variant and with flu-like syndrome at the onset; Subtype 3, both Th1 and Th2 cells-activated; Subtype 4, Th2 cell-skewed. This subtyping clearly indicated that T1D patients involve variable immune conditions.

### 3.3 *TYK2* Promoter Variant is Not Associated with Elevated IgE

Although we assessed the association of IgE levels and *TYK2* promoter genotype in T1D patients, we found no difference in IgE levels between *TYK2* promoter variant and wild type genotype (wild, median, 58.3 U/ml; variant, median, 45.8; *p* = 0.440) (Fig. S1).

### 3.4 T1D Patients with Flu-like Syndrome

Finally, we examined whether elevated IgE had any clinical significance in T1D patients with flu-like syndrome at the onset, of those patients suggestive of viral origin and related to *TYK2* promoter variant (Nagafuchi et al., 2015). In flu-like syndrome-associated patients, the major population was those with anti-GAD Ab negative and non-elevated IgE (*n* = 38; 50.0%, six *TYK2* promoter variants), belonging to the Subtype 2 population (Fig. S2, Table S1). When compared with the age-matched controls (Nagafuchi et al., 2015), only patients with anti-GAD Ab negative and non-elevated IgE, belonging to Subtype 2, had significantly higher incidence of *TYK2* promoter variant (OR, 4.22; 95%CI, 1.24–12.7; *p* = 0.011) (Table S1). Patients with elevated IgE were a minority of the T1D cohort (14.5%), and they did not associate with *TYK2* promoter variant even among them (Table S1). Thus, the observation seems to be consistent with our previous report that *TYK2* promoter variant was most likely associated with viral infections in diabetic patients dependent on defective type 1 IFN response (Nagafuchi et al., 2015), but not with Th2 immune response.

## 4 Discussion

In this study, we were able to show that T1D patients have overall elevated IgE and that their immune condition could be classified based on IgE and anti-GAD Ab profile into four subtypes. Although at the beginning of this study, we surmised that *TYK2* promoter variant may be associated with the pathogenesis of T1D modulating Th2 cell-dependent immunologic responses, the data we obtained contradicted this. *TYK2* promoter variant was not associated with elevated IgE (Fig. S1). In addition, Th1/Th2 ratio in diabetic patients did not exhibit any difference between patients with *TYK2* promoter variant and those with wild type genotype (Fig. S3). Although *TYK2* deficiency was reported to be the cause of HIES (Minegishi et al., 2006), recently, it was also reported that not all patients with *TYK2* deficiency presented elevation of IgE level as their phenotypes (Kreins et al., 2015). It was suggested that the important clinical phenotypes of *TYK2* deficiency is mainly mycobacterial and/or viral infections caused by impaired IL-12 and IFN-alpha/beta responses, but not elevated IgE levels (Kreins et al., 2015). Correspondingly, *TYK2* promoter variant presented a mild decrease of *TYK2* gene expressions with a mild reduction of IFN-induced anti-viral gene expressions (Nagafuchi et al., 2015). These observations suggested that *TYK2* promoter variant probably is not significant in Th2 type cell-dependent immunologic responses in diabetic patients. Because signal transducer and activator of transcription 3 (STAT3) and dedicator of cytokinesis 8 (DOCK8) are also reported as candidates for HIES (Engelhardt et al., 2009, Minegishi et al., 2007), these factors may possibly contribute to the elevated IgE in T1D patients.

Consistent with the classical concept of T1D, we confirmed that Subtype 1, which was considered to be a Th1 cell-activated state, was the major population even in IgE levels and anti-GAD Ab profile (Table 2). Accordingly, identification of the elevated IgE in T1D patients, though it was a minor population, has significance for further understanding of the immune condition and pathogenesis of T1D. Despite the suggestion that elevated IgE in T1D patients has a marked clinical significance, it was unclear whether elevated IgE is the cause or effect of T1D. Th2 cytokines exert their effects through direct and/or indirect mechanisms: they promote necrosis through occlusion of the microvasculature, stimulate activated T and B cells, enhance MHC class II expression, and amplify the cascade of anti-beta-cell immunity (Azar et al., 1999). IgE levels and anti-GAD Ab subtyping revealed the presence of a small but distinct population which was considered to be a Th2 cell-skewed immune condition as Subtype 4. Since IgE is a strong inducer of many inflammatory cytokines (Corry and Kheradmand, 1999), elevated IgE may possibly induce pancreatic beta-cell damage by mechanisms such as those described above. It was also reported that some diseases which are considered Th1 cell overactivation-associated diseases, including T1D, have mixed Th1/Th2 balanced conditions, and involve simultaneous Th1 and Th2 cell-activated phenotype (Kidd, 2003). We could detect the immune condition where both Th1 and Th2 cells were activated as Subtype 3. Subtypes 3 and 4 clearly showed that T1D not only have Th1 activated-immune status, but also have unique immune deviated condition, suggesting that all T1D cases can no longer be viewed as a Th1-associated disease (Azar et al., 1999, Østergaard et al., 2016).

Interestingly, anti-GAD Ab and IgE profile revealed that Subtype 2, anti-GAD Ab negative and non-elevated IgE, presented the second largest population, and was associated with *TYK2* promoter variant (Table 2). *TYK2* promoter variant did not affect IgE value, but it was most likely associated with viral infections in diabetic patients dependent on mildly defective IFN response (Nagafuchi et al., 2015). Consistently, patients with flu-like syndrome suggestive of viral origin mainly belonged to Subtype 2. Because positivity of autoantibody is decreased as time passed and *TYK2* promoter variant was not associated with anti-GAD Ab-positive patients (Nagafuchi et al., 2015), Subtypes 2 and 4 may contain patients who anti-GAD Ab turned to negative and have wild type *TYK2* gene. These populations could reduce the ratio of *TYK2* promoter variant in Subtype 2, while it still reaches statistical significance, suggesting that *TYK2* promoter variant serves as an important marker which characterizes Subtype 2. When we focused on patients with flu-like syndrome at T1D onset, positivity of anti-GAD Ab was lower (44.7%) than patients without flu-like syndrome at the onset (61.6%) (Table 1). Among them, the major population was anti-GAD Ab negative and low IgE, belonging to Subtype 2, and *TYK2* promoter variant also had a significantly high incidence (Table S1). On the other hand, classical anti-GAD Ab positive and low IgE subgroup was the second major group, and elevated IgE group was minimal. Thus, although T1D patients with flu-like syndrome were supposed to be very likely caused by the direct viral infection-mediated beta-cell damage without production of beta-cell-specific autoantibody, other types of T1D patients still occur, suggesting that viral infection may trigger the development of variable types of T1D, possibly dependent on the interplay between the pathogenicity of the virus and diverse immuno-reactivity among multiple host factors (Hober and Sauter, 2010).

In conclusion, we have reported that T1D could be subtyped into four groups based on anti-GAD Ab and IgE profile, as follows: Subtype 1, the major classical Th1 cell-dependent autoimmune T1D; Subtype 2, the second major group associated with *TYK2* promoter variant and maybe with virus-induced diabetes; Subtype 3, both Th1 and Th2 cells overactivated-immune condition; and Subtype 4, Th2 cell skewed-immune condition. Most importantly, only Subtype 2, the second major group, without anti-GAD antibody or elevation of IgE as described above, was associated with *TYK2* promoter variant, suggestive of deteriorated IFN response to resist against virus infections (Nagafuchi et al., 2015), and may be related to virus-induced diabetes, thereby enhancing the validity of subtyping of T1D as presented by this study.

We assessed *TYK2* promoter variant in YH and found that they had a rather high prevalence rate of *TYK2* promoter variant, compared with older-age-matched healthy controls, as reported previously (Table 1) (Nagafuchi et al., 2015). Since *TYK2* promoter variant serves as a higher risk for the development of diabetes (Nagafuchi et al., 2015), *TYK2* promotor variant positive young healthy people may develop diabetes along with aging, and thus the rate of *TYK2* promoter variant will become lower in older-aged people. Further age-dependent studies need to verify the hypothesis.

In the present study, while we studied a rather small number of patients and only in the Japanese population, we could subtype T1D based on anti-GAD Ab and IgE profile. Further studies of a larger scale, such as a worldwide study involving other ethnic groups with acute onset T1D, possibly associated with a flu-like viral syndrome, suggestive of viral origin, are needed to test our conclusion. In the future, Subtype 2 patients, suggestive of virus-induced diabetes, will be the target patients where an anti-diabetogenic virus vaccine will be able to protect diabetes-prone individuals if the screening had been done before the clinical onset of T1D. Thus, Anti-GAD Ab and IgE profiling is useful to realize the immune conditions underlying variable immuno-pathogenic mechanisms of T1D, and provide a clue to delineate the pathogenesis of T1D.

The following are the supplementary data related to this article.

Supplementary Method Th1/Th2 (IFN-γ x IL4/CD4) balance analysis.
Th1/Th2 balance (IFN-γ x IL4/CD4) was evaluated with flow cytometry (FACScan, Becton Dickinson, USA) in thirteen diabetic patients with wild type *TYK2* gene and eleven diabetic patients with *TYK2* promoter variant to examine the influence of *TYK2* promoter variant in Th1/Th2 balance. The test reagents were CD4PerCP and IFN-γ FITC/IL-4PE (Becton Dickinson, USA). This analysis was entrusted to LSI Medience Corporation, Japan. Image 1

Table S1 Characteristics of anti-GAD Ab, IgE, and *TYK2* promoter variant profile in T1D patients with flu-like syndrome at diabetes onset. Table S1

## References

[bb0005] American Diabetes Association (2014). Diagnosis and classification of diabetes mellitus. Diabetes Care.

[bb0010] Atkinson M.A., Eisenbarth G.S., Michels A.W. (2014). Type 1 diabetes. Lancet.

[bb0015] Azar S.T., Tamim H., Beyhum H.N., Habbal M.Z., Almawi W.Y. (1999). Type I (insulin-dependent) diabetes is a Th1- and Th2-mediated autoimmune disease. Clin. Diagn. Lab. Immunol..

[bb0020] de Beeck A.O., Eizirik D.L. (2016). Viral infections in type 1 diabetes mellitus - why the β cells?. Nat. Rev. Endocrinol..

[bb0025] Coppieters K.T., Boettler T., von Herrath M. (2012). Virus infections in type 1 diabetes. Cold Spring Harb. Perspect. Med..

[bb0030] Corry D.B., Kheradmand F. (1999). Induction and regulation of the IgE response. Nature.

[bb0035] De Amici M., Ciprandi G. (2013). The age impact on serum total and allergen-specific IgE. Allergy, Asthma Immunol. Res..

[bb0040] Engelhardt K.R., McGhee S., Winkler S., Sassi A., Woellner C., Lopez-Herrera G., Chen A., Kim H.S., Lloret M.G., Schulze I., Ehl S., Thiel J., Pfeifer D., Veelken H., Niehues T., Siepermann K., Weinspach S., Reisli I., Keles S., Genel F., Kutuculer N., Camcioǧlu Y., Somer A., Karakoc-Aydiner E., Barlan I., Gennery A., Metin A., Degerliyurt A., Pietrogrande M.C., Yeganeh M., Baz Z., Al-Tamemi S., Klein C., Puck J.M., Holland S.M., McCabe E.R.B., Grimbacher B., Chatila T.A. (2009). Large deletions and point mutations involving the dedicator of cytokinesis 8 (DOCK8) in the autosomal-recessive form of hyper-IgE syndrome. J. Allergy Clin. Immunol..

[bb0045] Hashiguchi T., Oyamada A., Sakuraba K., Shimoda K., Nakayama K.I., Iwamoto Y., Yoshikai Y., Yamada H. (2014). Tyk2-dependent bystander activation of conventional and nonconventional Th1 cell subsets contributes to innate host defense against Listeria monocytogenes infection. J. Immunol..

[bb0050] Haskins K., Cooke A. (2011). CD4 T cells and their antigens in the pathogenesis of autoimmune diabetes. Curr. Opin. Immunol..

[bb0055] Hober D., Sauter P. (2010). Pathogenesis of type 1 diabetes mellitus: interplay between enterovirus and host. Nat. Rev. Endocrinol..

[bb0060] (2015). IDF Diabetes Atlas Seventh Edition 2015.

[bb0065] Imagawa A., Hanafusa T. (2011). Fulminant type 1 diabetes-an important subtype in East Asia. Diabetes Metab. Res. Rev..

[bb0070] Izumi K., Mine K., Inoue Y., Teshima M., Ogawa S., Kai Y., Kurafuji T., Hirakawa K., Miyakawa D., Ikeda H., Inada A., Hara M., Yamada H., Akashi K., Niho Y., Ina K., Kobayashi T., Yoshikai Y., Anzai K., Yamashita T., Minagawa H., Fujimoto S., Kurisaki H., Shimoda K., Katsuta H., Nagafuchi S. (2015). Reduced Tyk2 gene expression in β-cells due to natural mutation determines susceptibility to virus-induced diabetes. Nat. Commun..

[bb0075] Jean-Baptiste V.S.E., Xia C.-Q., Clare-Salzler M.J., Horwitz M.S. (2017). Type 1 diabetes and type 1 interferonopathies: localization of a Type 1 common thread of virus infection in the pancreas. EBioMedicine.

[bb0080] Jun H.S., Yoon J.W. (2003). A new look at viruses in type 1 diabetes. Diabetes Metab. Res. Rev..

[bb0085] Kawasaki E., Eguchi K. (2004). Is type 1 diabetes in the Japanese population the same as among Caucasians?. Ann. N. Y. Acad. Sci..

[bb0090] Kidd P. (2003). Th1/Th2 balance: the hypothesis, its limitations, and implications for health and disease. Altern. Med. Rev..

[bb0095] Klamt S., Vogel M., Kapellen T.M., Hiemisch A., Prenzel F., Zachariae S., Ceglarek U., Thiery J., Kiess W. (2015). Association between IgE-mediated allergies and diabetes mellitus type 1 in children and adolescents. Pediatr. Diabetes.

[bb0100] Kounoue E., Izumi K.I., Ogawa S., Kondo S., Katsuta H., Akashi T., Niho Y., Harada M., Tamiya S., Kurisaki H., Nagafuchi S. (2008). The significance of T cells, B cells, antibodies and macrophages against encephalomyocarditis (EMC)-D virus-induced diabetes in mice. Arch. Virol..

[bb0105] Kreins A.Y., Ciancanelli M.J., Okada S., Kong X.-F., Ramírez-Alejo N., Kilic S.S., El Baghdadi J., Nonoyama S., Mahdaviani S.A., Ailal F., Bousfiha A., Mansouri D., Nievas E., Ma C.S., Rao G., Bernasconi A., Sun Kuehn H., Niemela J., Stoddard J., Deveau P., Cobat A., El Azbaoui S., Sabri A., Lim C.K., Sundin M., Avery D.T., Halwani R., Grant A.V., Boisson B., Bogunovic D., Itan Y., Moncada-Velez M., Martinez-Barricarte R., Migaud M., Deswarte C., Alsina L., Kotlarz D., Klein C., Muller-Fleckenstein I., Fleckenstein B., Cormier-Daire V., Rose-John S., Picard C., Hammarstrom L., Puel A., Al-Muhsen S., Abel L., Chaussabel D., Rosenzweig S.D., Minegishi Y., Tangye S.G., Bustamante J., Casanova J.-L., Boisson-Dupuis S. (2015). Human TYK2 deficiency: mycobacterial and viral infections without hyper-IgE syndrome. J. Exp. Med..

[bb0110] Leitner N.R., Witalisz-Siepracka A., Strobl B., Müller M. (2015). Tyrosine kinase 2 - Surveillant of tumours and bona fide oncogene. Cytokine.

[bb0115] Marroqui L., Dos Santos R.S., Fløyel T., Grieco F.A., Santin I., Op De Beeck A., Marselli L., Marchetti P., Pociot F., Eizirik D.L. (2015). TYK2, a candidate gene for type 1 diabetes, modulates apoptosis and the innate immune response in human pancreatic β-cells. Diabetes.

[bb0120] Minegishi Y., Saito M., Morio T., Watanabe K., Agematsu K., Tsuchiya S., Takada H., Hara T., Kawamura N., Ariga T., Kaneko H., Kondo N., Tsuge I., Yachie A., Sakiyama Y., Iwata T., Bessho F., Ohishi T., Joh K., Imai K., Kogawa K., Shinohara M., Fujieda M., Wakiguchi H., Pasic S., Abinun M., Ochs H.D., Renner E.D., Jansson A., Belohradsky B.H., Metin A., Shimizu N., Mizutani S., Miyawaki T., Nonoyama S., Karasuyama H. (2006). Human tyrosine kinase 2 deficiency reveals its requisite roles in multiple cytokine signals involved in innate and acquired immunity. Immunity.

[bb0125] Minegishi Y., Saito M., Tsuchiya S., Tsuge I., Takada H., Hara T., Kawamura N., Ariga T., Pasic S., Stojkovic O., Metin A., Karasuyama H. (2007). Dominant-negative mutations in the DNA-binding domain of STAT3 cause hyper-IgE syndrome. Nature.

[bb0130] Nagafuchi S., Kurisaki H., Katsuta H. (2013). Encephalomyocarditis Virus, in: Diabetes and Viruses.

[bb0135] Nagafuchi S., Kamada-Hibio Y., Hirakawa K., Tsutsu N., Minami M., Okada A., Kai K., Teshima M., Moroishi A., Murakami Y., Umeno Y., Yokogawa Y., Kogawa K., Izumi K., Anzai K., Iwakiri R., Hamaguchi K., Sasaki N., Nohara S., Yoshida E., Harada M., Akashi K., Yanase T., Ono J., Okeda T., Fujimoto R., Ihara K., Hara T., Kikuchi Y., Iwase M., Kitazono T., Kojima F., Kono S., Kurisaki H., Kondo S., Katsuta H. (2015). TYK2 promoter variant and diabetes mellitus in the Japanese. EBioMedicine.

[bb0140] Onengut-Gumuscu S., Chen W.-M., Burren O., Cooper N.J., Quinlan A.R., Mychaleckyj J.C., Farber E., Bonnie J.K., Szpak M., Schofield E., Achuthan P., Guo H., Fortune M.D., Stevens H., Walker N.M., Ward L.D., Kundaje A., Kellis M., Daly M.J., Barrett J.C., Cooper J.D., Deloukas P., Todd J.A., Wallace C., Concannon P., Rich S.S. (2015). Fine mapping of type 1 diabetes susceptibility loci and evidence for colocalization of causal variants with lymphoid gene enhancers. Nat. Genet..

[bb0145] Østergaard J.A., Laugesen E., Leslie R.D. (2016). Should there be concern about autoimmune diabetes in adults? Current evidence and controversies. Curr. Diab. Rep..

[bb0150] Scully T. (2012). Diabetes in numbers. Nature.

[bb0155] Shimada A., Maruyama T. (2004). Encephalomyocarditis-virus-induced diabetes model resembles “fulminant” Type 1 diabetes in humans. Diabetologia.

[bb0160] Shimoda K., Kato K., Aoki K., Matsuda T., Miyamoto A., Shibamori M., Yamashita M., Numata A., Takase K., Kobayashi S., Shibata S., Asano Y., Gondo H., Sekiguchi K., Nakayama K., Nakayama T., Okamura T., Okamura S., Niho Y., Nakayama K. (2000). Tyk2 plays a restricted role in IFN alpha signaling, although it is required for IL-12-mediated T cell function. Immunity.

[bb0165] Takeuchi O., Akira S. (2009). Innate immunity to virus infection. Immunol. Rev..

[bb0170] Wang Z., Zhang H., Shen X.-H., Jin K.-L., Ye G., Qian L., Li B., Zhang Y.-H., Shi G.-P. (2011). Immunoglobulin E and mast cell proteases are potential risk factors of human pre-diabetes and diabetes mellitus. PLoS One.

