# Supplementary material for: Subtyping of Type 1 Diabetes as Classified by Anti-GAD Antibody, IgE Levels, and Tyrosine kinase 2 (TYK2) Promoter Variant in the Japanese
Source: eBioMedicine. 2017 Aug 12;23:46–51. doi: 10.1016/j.ebiom.2017.08.012 (PMC5605380; doi:10.1016/j.ebiom.2017.08.012)
Supplement: Supplementary Method — Th1/Th2 (IFN-γ x IL4/CD4) balance analysis. Th1/Th2 balance (IFN-γ x IL4/CD4) was evaluated with flow cytometry (FACScan, Becton Dickinson, USA) in thirteen diabetic patients with wild type TYK2 gene and eleven diabetic patients with TYK2 promoter variant to examine the influence of TYK2 promoter variant in Th1/Th2 balance. The test reagents were CD4PerCP and IFN-γ FITC/IL-4PE (Becton Dickinson, USA). This analysis was entrusted to LSI Medience Corporation, Japan. [file mmc1.pdf]

Supplementary Method

Th1/Th2 (IFN-γ x IL4/CD4) balance analysis

Th1/Th2 balance (IFN-γ x IL4/CD4) was evaluated with flow cytometry (FACScan, Becton Dickinson, USA) in thirteen diabetic patients with wild type *TYK2* gene and eleven diabetic patients with *TYK2* promoter variant to examine the influence of *TYK2* promoter variant in Th1/Th2 balance. The test reagents were CD4PerCP and IFN-γ FITC/IL-4PE (Becton Dickinson, USA). This analysis was entrusted to LSI Medience Corporation, Japan.
