# Supplementary material for: Subtyping of Type 1 Diabetes as Classified by Anti-GAD Antibody, IgE Levels, and Tyrosine kinase 2 (TYK2) Promoter Variant in the Japanese
Source: eBioMedicine. 2017 Aug 12;23:46–51. doi: 10.1016/j.ebiom.2017.08.012 (PMC5605380; doi:10.1016/j.ebiom.2017.08.012)
Supplement: Table S1 — Characteristics of anti-GAD Ab, IgE, and TYK2 promoter variant profile in T1D patients with flu-like syndrome at diabetes onset. [file mmc2.docx]

| **Table S1. Characteristics of anti-GAD Ab, IgE, and *TYK2* promoter variant profile in T1D patients with flu-like syndrome at diabetes onset.** | | | | | | | | | |
| --- | --- | --- | --- | --- | --- | --- | --- | --- | --- |
|  | | | **T1D patients with flu-like syndrome^a^ at diabetes onset** | | | | | | |
|  |  |  |  |  | ***TYK2* promoter genotype** | | | | |
| **Belonging to Subtype-** | **Anti-GAD antibody (U/mL)** | **IgE (U/mL)** | **Number** |  | **Wild** | **Variant** | | **OR^b^ (95%CI^c^)** | ***p*-value** |
| 1 | ≧ 1.5 | < 170 | 27 |  | 25 | | 2 | 1.81 (0.189-8.59) | 0.344 |
| 2 | < 1.5 | < 170 | 38 |  | 32 | | 6 | 4.22 (1.24-12.7) | 0.011 |
| 3 | ≧ 1.5 | ≧ 170 | 7 |  | 6 | | 1 | 3.75 (0.077-34.4) | 0.274 |
| 4 | < 1.5 | ≧ 170 | 4 |  | 3 | | 1 | 7.44 (0.13-99.9) | 0.168 |
| Total (1-4) | - | - | 76 |  | 66 | | 10 | 3.42 (1.30-8.69) | 0.006 |
| Age-matched healthy control^d^ | | | 331 |  | 317 | | 14 | reference | reference |
| T1D, type 1 diabetes mellitus | | | | | | | | | |
| ^a^ Symptoms of flu-like syndrome include fever, chills, sore throat, muscle and joint aches, poor appetite, diarrhea, cough, and fatigue, suggestive of certain viral infections. | | | | | | | | | |
| ^b^ OR, odds ratio. | | | | | | | | | |
| ^c^ CI, confidence interval. | | | | | | | | | |
| ^d^ Data of age matched control were quoted from our previous study (Nagafuchi et al., 2015). | | | | | | | | | |
